# Intratracheal inoculation results in Brucella-associated reproductive disease in male mouse and guinea pig models of infection

**DOI:** 10.3389/fmicb.2022.1029199

**Published:** 2022-10-19

**Authors:** Martha E. Hensel, Lauren W. Stranahan, John F. Edwards, Angela M. Arenas-Gamboa

**Affiliations:** ^1^Department of Veterinary Pathobiology, Texas A&M University, College Station, TX, United States; ^2^MD Anderson Cancer Center, Michale E. Keeling Center for Comparative Medicine and Research, Bastrop, TX, United States

**Keywords:** *Brucella melitensis*, male, reproductive tract, vaccine, intratracheal inoculation

## Abstract

*Brucella* species are considered a significant cause of reproductive pathology in male and female animals. Importantly, *Brucella melitensis* can induce reproductive disease in humans. Reproductive pathogenesis and evaluation of newly developed countermeasures against brucellosis studies have traditionally utilized female animal models. However, any potential, new intervention for use in humans would need to be evaluated in both sexes. Therefore, animal models for male reproductive brucellosis are desperately needed to understand disease progression. Accordingly, we evaluated guinea pigs and mice using *B. melitensis* 16 M in an intratracheal model of inoculation at different stages of infection (peracute, acute, and chronic) with an emphasis on determining the effect to the male reproductive organs. Aerosol inoculation resulted in colonization of the reproductive organs (testicle, epididymis, prostate) in both species. Infection peaked during the peracute (1-week post-infection [p.i.]) and acute (2-weeks p.i.) stages of infection in the mouse in spleen, epididymis, prostate, and testicle, but colonization was poorly associated with inflammation. In the guinea pig, peak infection was during the acute stage (4-weeks p.i.) and resulted in inflammation that disrupted spermatogenesis chronically. To determine if vaccine efficacy could be evaluated using these models, males were vaccinated using subcutaneous injection with vaccine candidate 16 M*ΔvjbR* at 10^9^ CFU/100 μl followed by intratracheal challenge with 16 M at 10^7^. Interestingly, vaccination efficacy varied between species and reproductive organs demonstrating the value of evaluating vaccine candidates in multiple models and sexes. Vaccination resulted in a significant reduction in colonization in the mouse, but this could not be correlated with a decrease in inflammation. Due to the ability to evaluate for both colonization and inflammation, guinea pigs seemed the better model not only for assessing host-pathogen interactions but also for future vaccine development efforts.

## Introduction

Reproductive disease is a well-known consequence of brucellosis in animals. In small ruminants (sheep and goats), cattle, and dogs, infection during pregnancy typically results in abortions, stillbirths, and vertical transmission to the offspring (Garin-Bastuji and Blasco, 2016). Male animals of the same species may develop epididymitis and testicular degeneration, which can negatively impact fertility (Picard-Hagen et al., 2015; Garin-Bastuji and Blasco, 2016).

*Brucella* species can be divided into two groups based on structure of the lipopolysaccharide O chain: smooth or rough (Huddleson, 1943). While rough strains such as *B. canis* and *B. ovis* cause reproductive disease in dogs and sheep, respectively, only smooth strains (*B. melitensis* and *B. abortus*) have been documented to cause reproductive disease in both animals and men (Young, 1983, 1995; Corbel, 2006). *B. abortus* and *B. melitensis* infection in men may cause orchitis, epididymitis, and prostatitis (Young, 1983; Khan et al., 1989; Colmenero et al., 2007; Savasci et al., 2014).

Brucellosis is often spread from animals to humans through either direct contact with infected animals, inhalation of infectious aerosols, or indirectly through ingestion of unpasteurized milk (Corbel, 2006). Common symptoms regardless of sex are fever, inappetence, malaise, and joint pain (Young, 1995). Reproductive disease is a less common manifestation of disease, but retrospective studies in endemic areas estimate a range of 6.8–9.1% of genito-urinary issues in men are due to brucellosis (Yetkin et al., 2005; Colmenero et al., 2007; Gul et al., 2009).

It is important to assess the impact of the disease on both sexes and to understand potential differences associated with disease pathogenesis that may affect vaccine safety and efficacy and treatment performance in males. Historically, a majority of the comparative *in vivo* studies have been conducted in females with less known about the impact of brucellosis on the male reproductive tract (García-Carrillo, 1990; Grillo et al., 2012). While several studies in animal models for human disease (rhesus macaques, guinea pigs, and mice) have investigated the impact of smooth *Brucella* spp. on the male reproductive tract, these studies fail to fully characterize the kinetics or pathologic changes associated with infection (Hillaert et al., 1950; Mense et al., 2004; Izadjoo et al., 2008; Yingst et al., 2010).

The first step towards the goal of evaluating vaccines or therapeutics in males is to better characterize the effect of wild-type *Brucella* spp. on the male reproductive tract in commonly utilized animal models. For these studies, we elected to evaluate an aerosol exposure route, which is an important occupational hazard for certain professions including veterinarians, microbiological laboratorians, or abattoir workers (Young, 1983; Traxler et al., 2013). In order to deliver a targeted aerosol dose, an intratracheal route of inoculation using the PennCentury MicroSprayer™ was applied. This device has previously been used to inoculate guinea pigs and mice and generates a particle size that produces lower airway disease (Hensel et al., 2019, 2020). The benefit of intratracheal inoculation includes delivering a small particle size that is evenly distributed to the lower airways, and this route allows for a known infectious dose since it does not depend on the individual animal’s respiratory physiology such as respiratory rate and depth. Dose titration and kinetics of this route of inoculation for *B. melitensis* were previously characterized in female guinea pigs, which resulted in colonization and pathologic changes in the uterus and placenta (Gregory et al., 2019; Hensel et al., 2019, 2020). Therefore, the objective of this study was to first characterize the kinetics of *B. melitensis* 16 M infection with an emphasis on impact to the male reproductive tract following intratracheal inoculation in two of the most commonly used animal models, C57Bl/6 mice and Hartley guinea pigs. Following that, our objective was to compare the models in a practical application: evaluating vaccine efficacy in males.

## Materials and methods

### Bacterial strains

*B. melitensis* 16 M (originally isolated from an aborted goat fetus lung) was used in this study (Kahl-McDonagh et al., 2006). Vaccine candidate *B. melitensis 16 MΔvjbR* is a targeted gene deletion mutant derived from our laboratory stock (Arenas-Gamboa et al., 2008, 2012). The vjbR gene is a transcriptional regulator that influences expression of the type IV secretion system and contributes to virulence (Weeks et al., 2010). Bacteria were cultured on tryptic soy agar (TSA; Difco, Becton, Dickinson) at 37°C with 5% (vol/vol) CO_2_ for 72 h and harvested from plates with phosphate-buffered saline (PBS; Gibco). Using a Klett colorimeter to determine optical density, inoculums of either 1×10^7^ CFU/50 μl (guinea pig) or 1×10^7^ CFU/25 μl (mouse) were prepared. The inoculum dose was retrospectively verified through serial dilution and plating onto TSA medium in duplicate.

### Animal research ethics statement

All studies were performed with the approval of the Texas A&M University’s Institutional Animal Care and Use Committee (protocol: 2021–0038). Texas A&M University is fully accredited by the Association for Assessment and Accreditation of Laboratory Animal Care (AAALAC).

### Guinea pig infection with *Brucella melitensis* 16 M

Eighteen, 300–500 g (approximately 5 months old), male Hartley guinea pigs were obtained from Charles River (Wilmington, MA). Males were first assessed for reproductive capacity during an in-house breeding program and were then transferred to an ABSL-3 facility at Texas A&M University and housed individually in microisolator cages during experimental infection. After an acclimation period, animals were randomly divided into three inoculation groups (peracute, acute, chronic): 12 guinea pigs (*n* = 4/time point), which received *B. melitensis via* intratracheal inoculation, and 6 guinea pigs received sterile, endotoxin-free PBS as uninfected controls (*n* = 2/time point). Guinea pigs were anesthetized *via* intraperitoneal injection (i.p.) with a cocktail of ketamine (50 mg/kg) and xylazine (5 mg/kg). Once a surgical plane of anesthesia was achieved, animals were inoculated with 1×10^7^ CFU *B. melitensis* 16 M in 50 μl *via* intratracheal inoculation (IT) using the PennCentury MicroSprayer™ Aerosolizer (Wyndmoor, PA) as previously described (Gregory et al., 2019; Hensel et al., 2019, 2020). In brief, the guinea pig was placed in ventral-dorsal recumbency, and the larynx visualized using a small animal laryngoscope. The tip of the device was then placed in the proximal trachea. Negative control animals (*n* = 2 per time point) were sham inoculated with 50 μl of sterile, endotoxin-free PBS IT.

At peracute (2-weeks post-infection [p.i.]), acute (4-weeks p.i.), or chronic (8-weeks p.i.) time points, guinea pigs were euthanized i.p. with sodium pentobarbital (100 mg/kg) followed by cardiac exsanguination. One gram each of spleen, liver, lung, testicle, epididymis, and prostate were collected into pre-sterilized 2 ml collection tubes containing 1 ml PBS and 1.47 g of ceramic beads (Omni International). Tissues were homogenized as previously described using a Bead Ruptor Elite Bead Mill Homogenizer (Omni International), and homogenates were serially diluted and cultured on Farrell’s media. (Hensel et al., 2020) Following incubation for a minimum of 72 h, colonies were counted to determine CFU/g.

### Intratracheal inoculation of male C57Bl/6 mice

Twenty-eight, 8-10-week old, male C57BL/6 mice that had previously been used for an in-house breeding program were obtained from the Texas A&M Institute for Genomic Medicine. Males were transferred to an ABSL-3 facility at Texas A&M University and housed individually in microisolator cages during experimental infection. After an acclimation period, animals were randomly divided into four groups (peracute [1 and 2-weeks p.i.], acute [4-weeks p.i.], and chronic [8-weeks p.i.]): 20 mice (*n* = 5/time point) received *B. melitensis via* intratracheal inoculation, and 8 mice received sterile, endotoxin-free PBS as uninfected controls (*n* = 2/time point). Mice were anesthetized i.p. with ketamine (50 mg/kg) and xylazine (5 mg/kg) diluted in PBS, placed on a Mouse Intubation Platform (Penn-Century) in dorsoventral recumbency, and a small animal laryngoscope (Penn-Century) was used to visualize the larynx. The PennCentury MicroSprayer™ Aerosolizer was inserted into the proximal trachea and used to inoculate mice with 1×10^7^ CFU *B. melitensis* 16 M in 25 μl IT. Negative control animals (*n* = 2 per time point) were sham inoculated with 25 μl of sterile, endotoxin-free PBS IT.

At peracute (1 and 2-weeks p.i.), acute (4-weeks p.i.), or chronic (8-weeks p.i.) time points, mice were euthanized *via* carbon dioxide asphyxiation followed by cervical dislocation. Spleen, liver, lung, prostate, testicle, and epididymis were collected into 1 ml PBS. Tissues were weighed, homogenized, serially diluted, and plated as previously described (Stranahan et al., 2019). Following incubation for a minimum of 72 h, colonies were counted to determine CFU/g.

### Evaluation of histopathological changes in mice and guinea pigs

Testicle and epididymis from mice, and testicle, epididymis, and prostate from guinea pigs were collected at the aforementioned peracute, acute and chronic time points and fixed in 10% neutral buffered formalin (NBF; ThermoScientific) for a minimum of 48 h. Tissues were routinely processed, embedded in paraffin, sectioned at 5 μm, and stained with hematoxylin and eosin (H&E). Histologic changes of the testicle, epididymis, and prostate were scored for severity of inflammation (0–4), edema, necrosis, and tissue architecture changes by a board-certified anatomic veterinary pathologist as described in Supplementary Table S1.

### Immunohistochemistry to detect *Brucella* antigen

Five micrometer tissue sections of testicle/epididymis (mouse and guinea pig) and prostate (guinea pig only) were adhered to positively charged glass slides for immunohistochemistry. Slides were routinely processed, and antigen retrieval was performed as previously described using a 2,100 Antigen Retriever (Aptum Biologics Ltd. Southampton, UK; Hensel et al., 2019). Slides were blocked as previously described with Bloxall Blocking Solution (Vector Laboratories, Burlingame, CA) and normal goat serum (Vector Laboratories; Hensel et al., 2019). Primary incubation was performed overnight at 4^o^ C with a *Brucella* polyclonal rabbit antibody (Bioss Antibodies, Boston, MA) at dilution of 1:500. A Vectastain Elite® ABC HRP Kit (Vector Laboratories) with an avidin/biotinylated anti-rabbit secondary antibody was used according to the manufacturer’s instructions. Antigen was visualized with a Betazoid DAB chromagen kit (Biocare Medical, Pachecho, CA). The slides were counterstained with Gills’s hematoxylin III and cover slipped.

### Comparison of the mouse and guinea pig as models to assess vaccine efficacy

Five, 6–8 week old, male C57BL/6 mice and four, 400 g male Hartley guinea pigs were vaccinated subcutaneously with 16 M*ΔvjbR* at 1×10^9^ CFU/100 μl and then rested for 6-weeks. Vaccinated animals were then moved to an ABSL-3 and housed as a group (mice) or individually (guinea pigs) in microisolator cages. Challenge inoculum of 1×10^7^ CFU 16 M *B. melitensis* was prepared, and animals were anesthetized and inoculated IT as described above. At 1-week post-challenge, mice were euthanized *via* CO_2_ asphyxiation followed by cervical dislocation, and spleen, liver, lung, testicle, epididymis, and prostate were collected for culture. At 2-weeks post-challenge, guinea pigs were euthanized as previously described, and the same tissues were collected for culture on Farrell’s media. Spleen, liver, lung, testicle, and epididymis were also collected for histopathology.

### Statistical analysis

Statistical analysis of infection kinetics in the mouse and guinea pig was performed using two-way analysis of variance (ANOVA) followed by Šídák’s multiple comparisons to evaluate differences in organ colonization by time point. The limit of detection using standard culture methods is 10 CFU/g; as such, several tissues were reported as 0 indicating colonization was less than 10 CFU/g. Therefore, to evaluate the normality of the data, Q-Q plots were assessed following 2-way ANOVA. The sum of the histologic lesion scores between time points and negative controls were evaluated by the Kruskal–Wallis test followed by Dunn’s multiple comparisons. Analysis of vaccine efficacy was performed using multiple Mann Whitney U Test with Šídák-Bonferroni correction to compare colonization following challenge in vaccinated and unvaccinated mice and guinea pigs. All tests were performed using GraphPad Prism v9 (GraphPad Software, San Diego, CA).

## Results

The first objective was to characterize intratracheal inoculation with *B. melitensis* 16 M in male C57BL/6 mice and Hartley guinea pigs and characterize the effects of infection, not only on the lung and hematopoietic targets, but most importantly in the reproductive tract. Infertility, orchitis, and epididymitis in males has been reported in naturally infected males, both human and animal, but efforts to develop a small animal laboratory model for reproductive disease have been sporadic (Young, 1983; Corbel, 2006; Izadjoo et al., 2008). Aerosol inoculation is an important route of natural transmission in people, but this inoculation route has not been evaluated for male reproductive disease (Pappas et al., 2003, 2006). To assess common milestones of disease, male animals were euthanized at peracute (1 to 2-weeks p.i.), acute (4-weeks p.i.), or chronic (8-weeks p.i.) phases of infection. In humans, these stages are characterized by the onset of fever and flu-like symptoms during the peracute and acute stages while reproductive disease is often identified in the chronic stages when clinical signs such as scrotal swelling and pain develop (Young, 1983).

**Figure 1:**
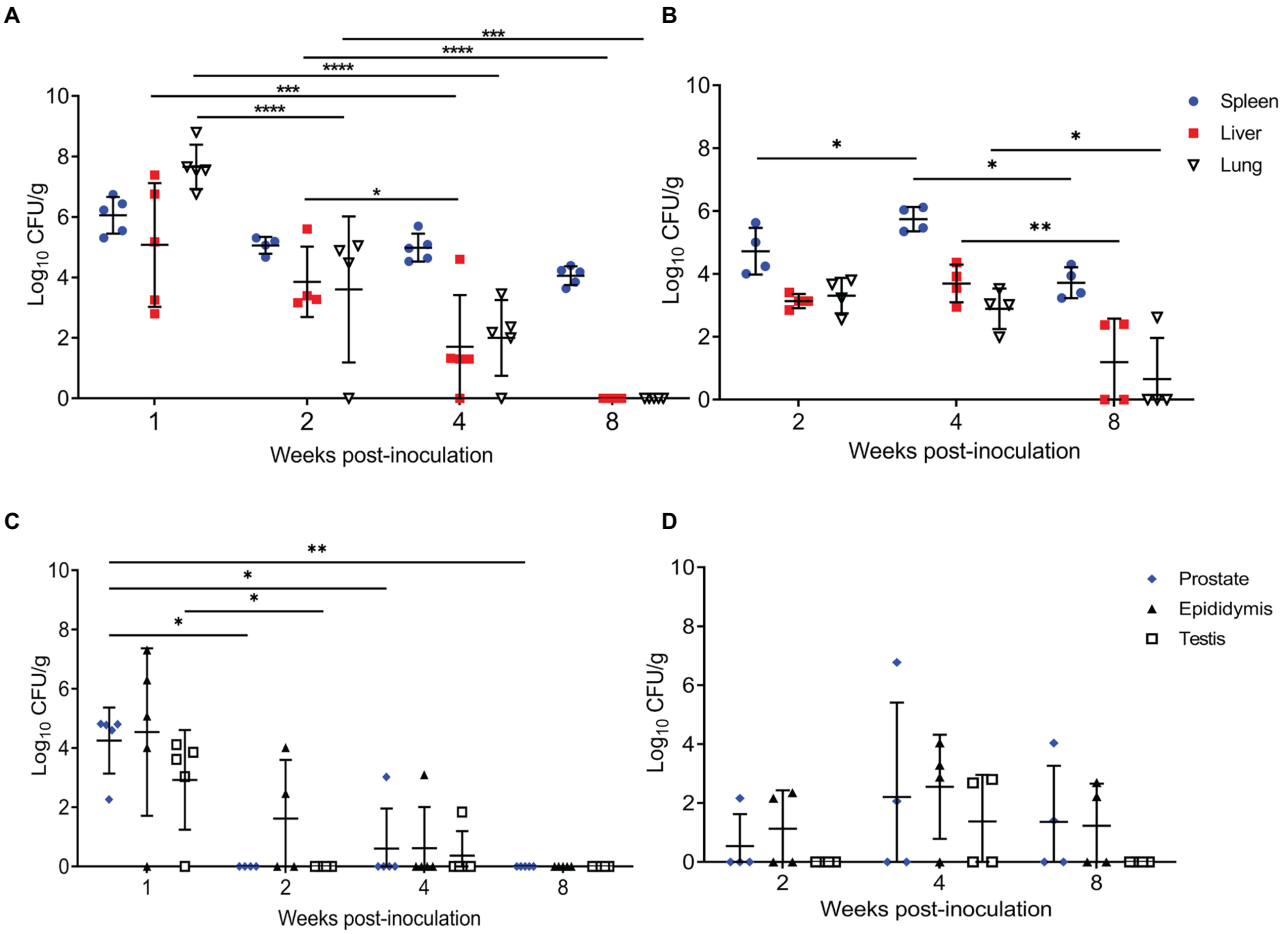
Intratracheal inoculation results in colonization of the spleen, liver, lung, and reproductive organs in mice and guinea pigs. **(A)** Spleen, liver, and lung were collected from mice (*n* = 5/time point) inoculated with 1×10^7^ CFU/25 μl *via* intratracheal inoculation and euthanized at 1-,2-,4-, and 8-weeks post-challenge. **(B)** Spleen, liver, and lung were collected from guinea pigs (*n* = 4/time point) inoculated with 1×10^7^ CFU/50 μl *via* intratracheal inoculation and euthanized at 2-,4-, and 8-weeks post-challenge. Epididymis, testicle, and prostate were collected at the same time points in mice **(C)** and guinea pigs **(D)**. Differences in mean colonization between time points analyzed by 2-way ANOVA followed by Šídák’s multiple comparison test. Horizontal bar indicates mean colonization. Results reported as log_10_ CFU/g with S.D. **p < 0.01, ***p < 0.001, ****p < 0.0001.

To determine if IT inoculation resulted in systemic infection, the spleen, liver, and lung were cultured. In the 2-week mouse group, an anesthetic death occurred during intratracheal inoculation. Organ colonization of the spleen was detected in 100% of mice at the peracute time points of 1-week p.i. (5/5) and 2-weeks p.i. (4/4) and 100% of guinea pigs (4/4) by 2-weeks p.i. (Figures 1A,B). At 1-week p.i., 100% of mice and guinea pigs had colonization of the lungs confirming intratracheal inoculation resulted in infection of the lung. Colonization of the spleen, liver, and lung peaked at 1-week p.i. in the mice and was significantly increased at 1-week p.i. compared to 2-,4-, and 8-weeks p.i. (Figure 1A). No significant differences were detected between 2- and 4-weeks p.i. in mice in the spleen, but colonization of the liver (*p* < 0.05) and lung (*p* < 0.001) at 4-weeks p.i. were significantly decreased compared to 2-weeks p.i. The kinetics of colonization mimics that seen in other aerosol models with *Brucella melitensis* in mice in which colonization peaks during peracute infection and declines by 4-weeks p.i. (Mense et al., 2001; Kahl-McDonagh et al., 2007).

Guinea pigs were evaluated at 2-, 4-, and 8-weeks p.i. since previous experiments utilizing IT inoculation of guinea pigs have demonstrated that infection required at least 2-weeks to become established (Hensel et al., 2019, 2020). At the peracute stage of infection (2-weeks p.i.), 100% of the guinea pigs had colonization of the spleen, liver, and lung (Figure 1B). Colonization of male guinea pigs is similar to that seen in non-pregnant and pregnant female guinea pigs when dosed with 10^7^ IT, where colonization of the spleen, liver, and lung occurs in 91.6% of the animals during the peracute stage of infection and 100% by the acute stage (Hensel et al., 2019, 2020). When the kinetics of colonization in guinea pigs was explored, colonization of the liver (*p* < 0.01), spleen (*p* < 0.05), and lung (*p* < 0.05) was significantly increased at 4-weeks compared to 8-weeks p.i. In contrast to mice, infection in the guinea pigs required 4-weeks p.i. to peak in the spleen and liver. Whereas colonization in lung peaks in mice at 1-week p.i. and declines exponentially thereafter, colonization in the guinea pig was stable with no significant difference in mean CFU/g between 2- and 4-weeks p.i. (Figure 1B). However, colonization of lung (*p* < 0.05) significantly decreased from 4- to 8-weeks p.i. (Figure 1B).

Several studies have been conducted in male guinea pigs and mice using aerosol routes of inoculation, but the reproductive organs were not evaluated (Elberg and Henderson, 1948; Druett et al., 1956; Henning et al., 2012). Therefore, a second objective was to determine if IT inoculation would generate reproductive disease in males. Interestingly, the reproductive organs (prostate, testicle, epididymis) in both mice and guinea pigs were colonized following IT inoculation with 10^7^ CFU *B. melitensis* (Figures 1C,D). Like the pattern seen in the other organs, infection peaked at 1-week p.i. in the mouse and at 4-weeks p.i. in the guinea pig. Colonization did not persist in the mouse beyond the acute stage of infection (4-weeks p.i.); however, colonization of the epididymis, testicle, and prostate persisted in 50% of the guinea pigs through 4-weeks, and in the epididymis and prostate of 50% (2/4) at 8-weeks p.i. (Figures 1C,D). While colonization was not significantly different when compared to controls or by time points in the guinea pigs, these results suggest that IT inoculation can induce chronic disease of the reproductive organs.

**Figure 2:**
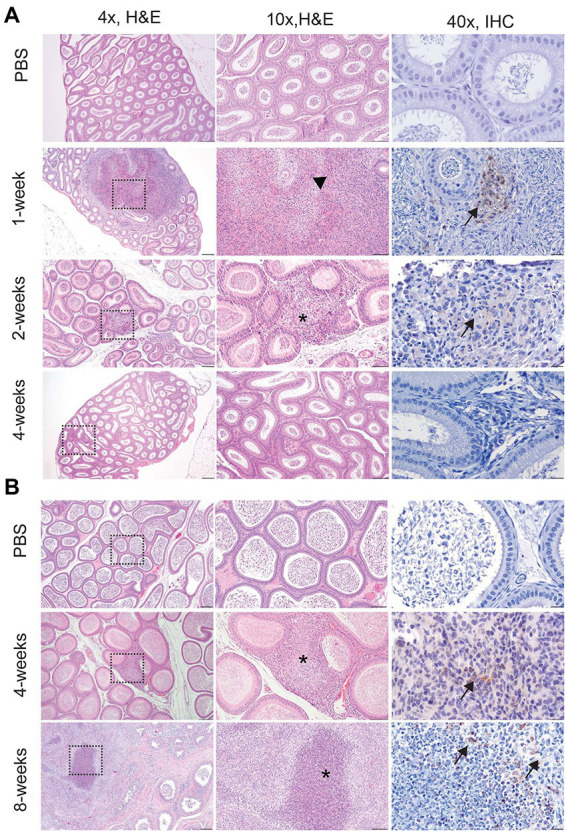
Infection with *Brucella melitensis* results in epididymitis at the peracute stage of infection in mice and the acute and chronic stages in guinea pigs. Mice **(A)** and guinea pigs **(B)** were inoculated with 10^7^ *B. melitensis via* IT inoculation, and the epididymis was collected for histology. Representative images for the mouse **(A)** at the peracute (1- and 2-week p.i.) and acute (4-week p.i.) and guinea pig **(B)** at the acute (4-week p.i.) and chronic (8-weeks p.i.) stages of infection. A single mouse had rupture of the epididymal duct with intense inflammatory infiltrate (arrowhead). Macrophages in this region had abundant intracytoplasmic bacteria (IHC, arrows). The lesion in mice at 2-weeks and guinea pigs at 4-weeks consisted of a focally extensive infiltrate of histiocytes and neutrophils (*) in the interstitium of the epididymal duct. Scant macrophages were positive for *Brucella* antigen by IHC (arrows). By 8-weeks p.i., guinea pigs developed spermatic granulomas from rupture of the epididymal duct, and macrophages in the lesion contained abundant intracytoplasmic and extracellular *Brucella* antigen (IHC, arrows). Tissues were stained with hematoxylin and eosin (H&E) and a polyclonal *Brucella* antibody (IHC) at 1:500 with Gill’s hematoxylin counterstain. Dashed box of 4x image indicates area highlighted for the 10x and 40x images. 4x, scale bar = 100 μm; 10x, scale bar = 50 μm; 40x, scale bar = 10 μm.

**Figure 3:**
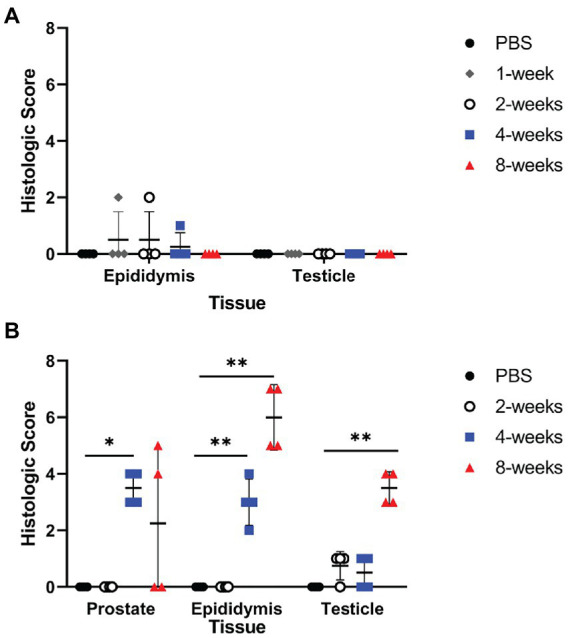
Infection resulted in significant inflammation in the epididymis and prostate of guinea pigs. Histologic changes of the testicle, epididymis, and prostate were scored for inflammation, edema, necrosis, and tissue architecture changes in the mouse **(A)** and guinea pig **(B)**. Prostate from the mouse was not available for histologic examination. Histologic scores between PBS controls and infected animals at each time point were compared using the Kruskal-Wallis test followed by Dunn’s multiple comparisons. Horizontal line indicates median. Statistical significance compared to controls *p < 0.05; **p < 0.01.

Culture results do not provide the full picture of the impact of infection upon the reproductive organs; therefore, testicle, epididymis, and prostate from guinea pigs and testicle and epididymis from mice were evaluated for changes in tissue architecture, inflammation, and effect on spermatogenesis (Supplementary Table S1). *B. melitensis* causes epididymitis, prostatitis, and testicular swelling in naturally infected small ruminants and in humans (Boyd, 1938; Young, 1983; Khan et al., 1989; Yetkin et al., 2005; Corbel, 2006; Savasci et al., 2014). Epididymitis is a common lesion that results from infection; therefore, histologic sections of epididymis were examined to correlate colonization with microscopic evidence of disease (Khan et al., 1989; Gul et al., 2009; Savasci et al., 2014). During the peracute stage of infection, 25% (1/4) of the mice had epididymitis characterized by degeneration and rupture of the epididymal duct which generated an intense histiocytic reaction to the extra-tubular spermatids (Figure 2A). Degeneration and rupture of the epididymal duct was presumably due to infection with *B. melitensis* as macrophages in the lesion contained abundant, intracytoplasmic bacteria that stained positively with a polyclonal *Brucella* antibody (Figure 2A). Despite a level of colonization considered to be too low to be detected by culture (<10 CFU/g) in the mouse, the epididymis had evidence of an inflammatory infiltrate of macrophages in the epididymal duct interstitium at 2-weeks p.i. (Figure 2A). By 4-weeks p.i., the epididymis had no detectable lesions, but cross-sections of the epididymal duct subjectively appeared to have fewer spermatids (Figure 2A). In the mouse, the mean histologic score was not statistically increased compared to uninfected controls (Figure 3A).

Colonization and lesion development were delayed in guinea pigs, but the histologic lesions were more pronounced. At 4-weeks p.i., mean colonization in the epididymis was 2.6 logs with a mean histologic score of 3 (*p* < 0.01) compared to uninfected controls (Figure 3B). Similar to the mouse during the acute stage of infection, the histologic lesions at 4-weeks consisted of multifocal inflammatory infiltrates of macrophages and neutrophils in the interstitial tissue of the epididymal duct (Figure 2B). However, by 8-weeks p.i., colonization was 1.1 logs, but the mean histologic score was significantly increased (*p* < 0.01) compared to 4-weeks p.i. and PBS controls (*p* < 0.01; Figure 3B). At 8-weeks p.i., the epididymal changes consisted of multifocal to coalescing necrotizing and histiocytic epididymitis with no mature spermatids in the epididymal duct which suggests spermiostasis (Figure 2B). The lack of spermatids within the epididymal duct was likely secondary to disordered spermatogenesis in the testicle. When a polyclonal anti-*Brucella* antibody was applied to sections, epididymal lesions at 4- and 8-weeks p.i. had abundant intralesional *Brucella* antigen within foci of necrosis, and macrophages contained intracytoplasmic antigen (Figure 2B).

**Figure 4:**
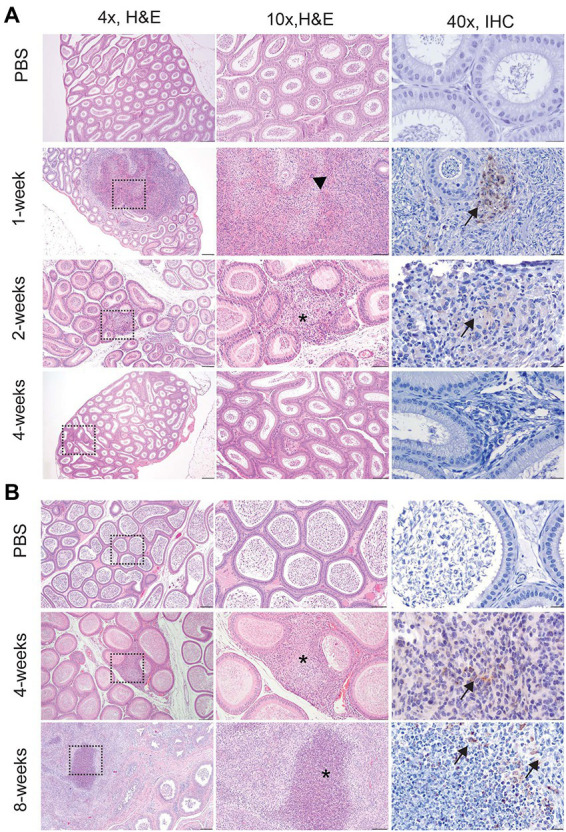
Infection with 16 M *B. melitensis* disrupts spermatogenesis in the guinea pig. Testicle from mice **(A)** at the peracute (1- and 2-week p.i.) and acute (4-week p.i.) and guinea pigs **(B)** at the acute (4-week p.i.) and chronic (8-weeks p.i.) stages of infection following IT inoculation with 10^7^ *B. melitensis*. No lesions were detected in the mouse at any stage of infection. The lesion in guinea pigs at 8-weeks consisted of a focally extensive area of necrosis with macrophages and neutrophils (yellow arrow). Adjacent seminiferous tubules were degenerate (*) with disrupted spermatogenesis. Macrophages were positive for *Brucella* antigen by IHC (black arrow). Tissues were stained with hematoxylin and eosin (H&E) and a polyclonal *Brucella* antibody (IHC) at 1:500 with Gill’s hematoxylin counterstain. Dashed box of 4x image indicates area highlighted for the 10x and 40x images. 4x, scale bar = 100 μm; 10x, scale bar = 50 μm; 40x, scale bar = 10 μm.

Orchitis (inflammation of the testicle) is less common in animals than epididymitis, but the literature reporting disease in men does not often distinguish between primary testicular or epididymal infection and instead describes the lesion as epididymo-orchitis (Khan et al., 1989; Colmenero et al., 2007; Gul et al., 2009). Despite colonization of testicle in the mouse, no histologic evidence of disease was detected at any time point (Figure 4A). In guinea pigs, the earliest lesion was high-protein edema that expanded the interstitium and separated the seminiferous tubules (Figure 4B). This lesion did not appear to impact spermatogenesis because maturation of spermatogonia was orderly and mature spermatids were in the epididymal duct. Colonization did not correlate with inflammation in the testicle in either species. Although no bacteria were recovered at 8-weeks p.i. from the testicle, guinea pigs had evidence of diminished and disordered spermatogenesis at the chronic stage of infection (Figure 4B). A single guinea pig had evidence of on-going inflammation characterized by focal necrosis of the seminiferous tubules surrounded by an intense inflammatory reaction composed of neutrophils and macrophages (Figure 4B). It is likely that infection at an earlier time point led to necrosis of the tubules and a localized inflammatory reaction to the release of “foreign” material of immature spermatozoa.

**Figure 5:**
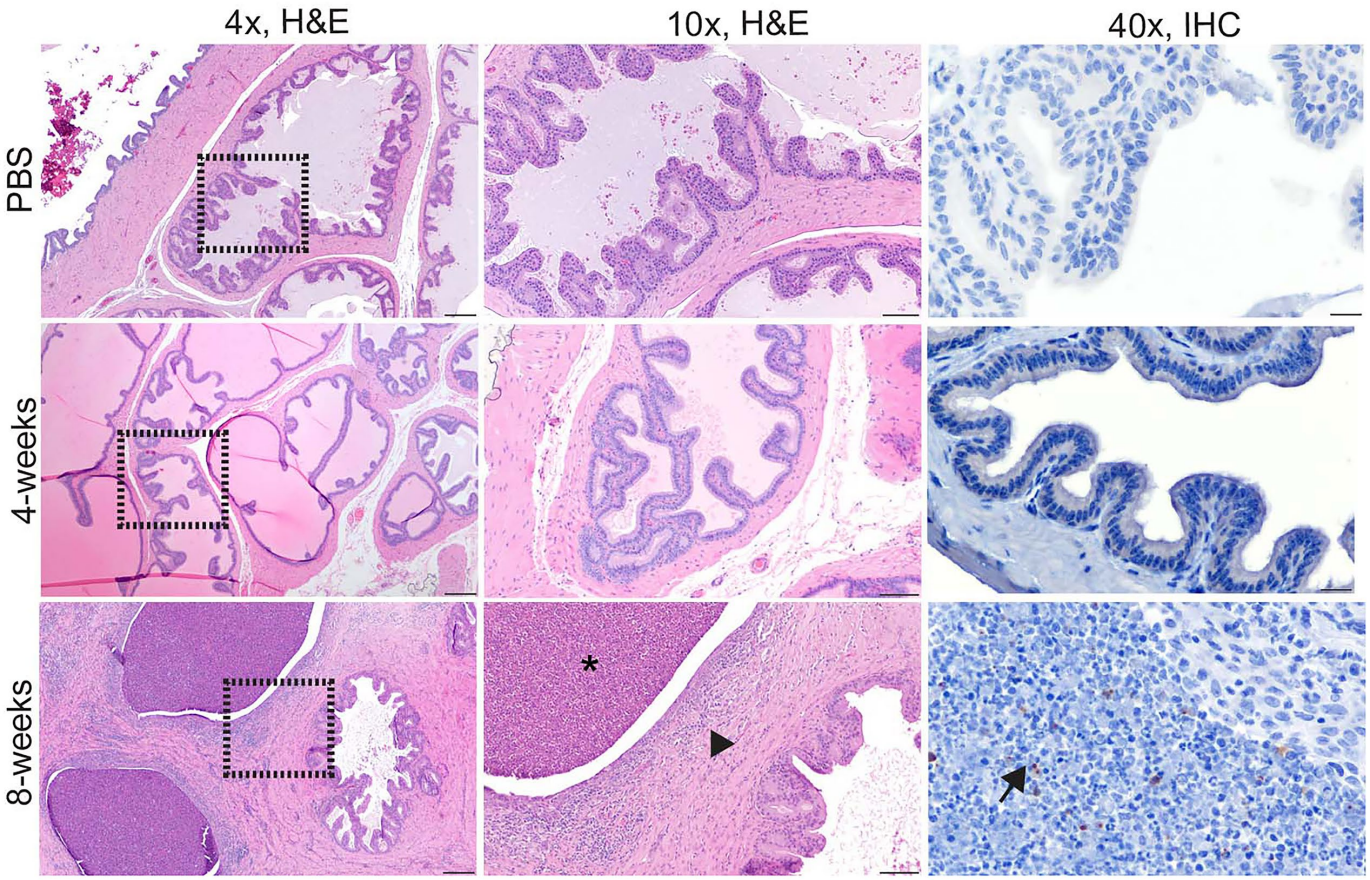
Intratracheal inoculation with 16 M *B. melitensis* in the guinea pig results in prostatitis at the chronic time point. Prostate from guinea pigs at the acute (4-week p.i.) and chronic (8-weeks p.i.) stages of infection following IT inoculation with 10^7^ *B. melitensis*. Two guinea pigs in the chronic group had abundant fibrosis separating the glands (arrowhead) with infiltrates of lymphocytes and plasma cells. The prostatic glands were dilated and contained abundant degenerate neutrophils and cellular debris (*). IHC of the prostate lesion revealed extracellular *Brucella* antigen within the area of cellular debris (arrow). Tissues were stained with hematoxylin and eosin (H&E) and a polyclonal *Brucella* antibody (IHC) at 1:500 with Gill’s hematoxylin counterstain. Dashed box of 4x image indicates area highlighted for the 10x and 40x images. 4x, scale bar = 100 μm; 10x, scale bar = 50 μm; 40x, scale bar = 10 μm.

The prostate is an accessory sex organ responsible for producing part of the seminal fluid (Foley, 2001; Motrich et al., 2018). In men, prostatitis is reported to occur with infection (Boyd, 1938; Young, 1983). Due to the small organ size of the prostate in the mouse, culture was prioritized over histology, which prevented any correlation of colonization in this species. Culture was prioritized as it is the gold standard of determining infection and can be used to quantify viable organisms. Colonization of the prostate was detected in 2 of 4 (50%) guinea pigs at the acute stage of infection (4-weeks p.i.), and chronic time point (8-weeks p.i.). Inflammatory lesions were noted in the prostate at 4-weeks p.i. and 8-weeks p.i. which is reflected by a significant increase in mean histologic score (Figure 3B). The lesion was characterized by necrosis of the epithelium of the prostate acini with intense neutrophil and histiocyte coagulum replacing the normal seminal fluid (Figure 5). Acini were surrounded and separated by thick bands of fibrosis indicating chronic inflammation and tissue remodeling (Figure 5). *Brucella* antigen was detected by IHC within the foci of necrosis and intracellularly within macrophages (Figure 5). The lack of detectable colonization suggests the positive IHC response was due to dead bacteria contained within areas of necrosis and macrophages. The mismatch between colonization and histologic score in the epididymis and prostate suggests that *B. melitensis* can induce an intense inflammatory response in the absence of significant colonization.

**Figure 6:**
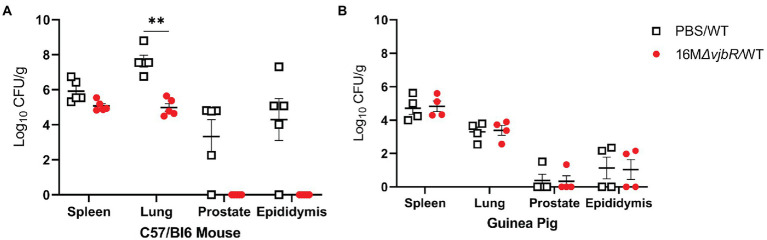
Vaccination in mice reduced colonization in the spleen, lung, and reproductive tissues. Mice and guinea pigs were vaccinated with 1×10^9^ CFU/100 μl *B. melitensis* 16 M*ΔvjbR* (16 M*ΔvjbR*/WT) subcutaneously or sham vaccinated with 100 μl endotoxin-free PBS (PBS/WT) and were challenged IT with 1×10^7^ CFU 16 M *B. melitensis*. **(A)** Mice (*n* = 5) were euthanized 1-week post challenge and spleen, lung, epididymis, and prostate were cultured on Farrell’s media. **(B)** Spleen, lung, epididymis, and prostate were collected from guinea pigs (*n* = 4) at 2-weeks post-challenge and cultured on Farrell’s. Differences in colonization between groups analyzed by multiple Mann Whitney U Test with Sidak-Bonferroni correction. Horizontal bar indicates mean. Results reported as log_10_ CFU/g with S.D. **p < 0.01.

After establishing that IT inoculation results in male reproductive disease in both species, the next objective was to evaluate a vaccine candidate to compare efficiency of the models. Since the goal was to establish the relative usefulness of the model rather than to evaluate the vaccine candidate, a reference strain was not used for comparison. In the vaccinated mouse group, an anesthetic death occurred during intratracheal inoculation. When vaccinated mice were challenged with 16 M *B. melitensis*, only the lung had a statistically significant reduction in mean colonization compared to unvaccinated animals challenged with 16 M (Figure 6A). While not statistically significant, vaccination reduced colonization in the epididymis and prostate to below the limit of detection by culture (<10 CFU/g). Unexpectedly, vaccination did not reduce colonization in the guinea pigs (Figure 6B). This contrasts with a previous experiment in which pregnant guinea pigs vaccinated with 16 M*ΔvjbR* were protected following challenge. (Hensel et al., 2020) It is possible that 16 M*ΔvjbR* requires an adjuvant to increase the efficacy; in the pregnant guinea pig challenge model, the vaccine was administered with Quil-A (Hensel et al., 2020). The contrasting results in male mice and guinea pigs and between female and male guinea pig stresses the value of evaluating novel vaccine candidates in more than one model and using both sexes.

## Discussion

*B. abortus* and *B. melitensis* cause reproductive disease in both males and females, making it critical to be able to model disease in both sexes to better understand disease pathogenesis underlying reproductive disease as well as to evaluate newly developed countermeasures (i.e., vaccines and therapeutics) for use in humans.

*Brucella*-associated reproductive disease has been evaluated in female mice and more recently in female guinea pigs (Tobias et al., 1993; Kim et al., 2005; Grillo et al., 2012; Byndloss et al., 2019; Hensel et al., 2019, 2020). While vaccination strategies in production animals focus on vaccinating female animals to prevent the spread of disease to other animals and humans, any vaccine for use in humans would need to be safe and efficacious in both sexes to be useful in preventing disease. In endemic regions, infection with *B. melitensis* results in scrotal swelling, pain, epididymitis, and orchitis in men (Khan et al., 1989; Yetkin et al., 2005; Colmenero et al., 2007; Gul et al., 2009). This study aimed to provide a foundation for exploring reproductive brucellosis in men through comparative animal models: mice and guinea pigs.

Hartley guinea pigs and C57BL/6 mice have both been used extensively in *Brucella* spp. vaccination and pathogenesis studies (García-Carrillo, 1990; Grillo et al., 2012). Guinea pigs were previously the model of choice to assess virulence and commercially available vaccines for brucellosis were tested in guinea pigs (Huddleson, 1943; García-Carrillo, 1990). Previous studies in male mice and guinea pigs have utilized intraperitoneal, intratesticular, or intra-gastric (oral) routes of inoculation to evaluate the impact of the male reproductive organs (Meyer et al., 1922; Hillaert et al., 1950; Moulton and Meyer, 1958; Jimenez de Bagues et al., 1993; Izadjoo et al., 2008). When considering a route of inoculation for experimental use, it is important to consider the relevance to natural transmission pathways as well as the anatomy and physiology of the animal. I.p. inoculation is artificial route of inoculation and is especially challenging for studies investigating the impact on the male reproductive system because the scrotum and peritoneal cavity are continuous; therefore, the inoculum can move directly to the reproductive tissues without first generating systemic disease/bacteremia (Knoblaugh et al., 2018a). Aerosol or oral routes of inoculation are most applicable for studies investigating natural transmission methods for *Brucella* spp. in humans (Young, 1983; Corbel, 2006).

For this study, intratracheal inoculation was utilized as it mimics a natural transmission route, and IT intratracheal inoculation with 10^7^ 16 M has been shown to reliably produce reproductive disease in female guinea pigs (Hensel et al., 2019, 2020). Although a dose of 10–100 CFU reportedly results in clinical symptoms in humans, occupational exposures such as handling aborted placentas or unknown microbial cultures on an open bench could result in a much higher aerosolized dose (Pappas et al., 2003, 2006). Furthermore, previous work using IT inoculation in guinea pigs and an aerosolization study in rhesus macaques both demonstrated that 10^3^ CFU was the minimum dose required to generate disease; however, in both rhesus macaques and guinea pigs, higher doses (10^5^ to 10^6^ CFU, respectively) resulted in a higher percentage of infected animals and generated reproductive disease (Mense et al., 2004; Hensel et al., 2019). Therefore, a higher dose was administered *via* IT inoculation in mice and guinea pigs to determine the impact on the reproductive organs.

In the male mice, colonization initially occurred in the reproductive tissues at higher levels than that seen in the guinea pigs, but inflammation was more severe in the guinea pigs. This suggests that guinea pigs are better at replicating the natural course of infection because they develop lesions in the epididymis and testes, which impact spermatogenesis. A study by Izadjoo et al. in C57BL/6 male mice found that oral inoculation with 10^11^ *B. melitensis* resulted in low levels of infection in the testicle starting 2-weeks p.i. through 8-weeks p.i.(Izadjoo et al., 2008). This infection was accompanied by perivascular inflammation of the epididymis, but no lesions were reported in the parenchyma of the testicle or epididymis (Izadjoo et al., 2008). Taken together, the Izadjoo study and our current results suggest that male mice do not develop inflammation in the reproductive organs following oral or aerosol inoculation with *B. melitensis* despite evidence of colonization (Izadjoo et al., 2008). These results also stress the importance of correlating colonization with histologic evidence of disease.

Early studies utilizing artificial routes of intraperitoneal or intratesticular inoculation demonstrated that guinea pigs develop abscesses of the testicle and epididymis when infected with *B. suis, abortus,* and *melitensis* (Meyer et al., 1922; Hillaert et al., 1950; Braude, 1951; Moulton and Meyer, 1958). When male guinea pigs were used in the early aerosol exposure research, these studies did not characterize the effect on the male reproductive tract (Elberg and Henderson, 1948; Harper, 1955; Druett et al., 1956). Therefore, it was unknown if aerosol transmission would generate reproductive pathology in the guinea pig model. Interestingly, guinea pigs inoculated intratracheally developed lesions in the parenchyma of the reproductive organs even with limited colonization at the chronic stage of infection. In this study, evidence indicates an intratracheal dose of 10^7^ generates acute and chronic infection accompanied by significant inflammation.

*B. melitensis* and *B. suis* have been used in aerosol studies in rhesus macaques as a model for human brucellosis, which have demonstrated that an aerosol dose of 10^5^ CFU *B. melitensis* and 10^7^ CFU *B. suis* could induce orchitis and epididymitis in a small number of animals (Mense et al., 2004; Yingst et al., 2010). Mense et al. detected histologic lesions of infection in a single animal 63-days post-inoculation with 10^5^ which resemble the lesions described in guinea pigs of this report. The study by Yingst et al. confirmed infection by polymerase chain reaction (PCR) rather than culture. Therefore, the reported lesion in this case may not have been due to active colonization. The lesions reported in the macaques are like those we describe in the guinea pig. Thus, it may be possible to have active inflammation in the absence of positive culture from the tissue. Additional studies are required to elucidate this seeming contradiction of active inflammation without a detectable agent.

In natural hosts such as bulls, rams, bucks, and dogs the histologic lesion is characterized by necrosis, fibrosis, and atrophy of the testicle and epididymis, which is replicated in both the mouse and guinea pig (Lambert et al., 1963; Greene and Carmichael, 2012; Foster, 2016). In humans, the diagnosis is often by serology and response to antibiotic therapy rather than histologic evaluation. Therefore, we cannot definitively know if the guinea pig or mouse reflect the underlying pathology (Young, 1983; Colmenero et al., 2007). However, the severity of the clinical symptoms in infected men (testicular swelling/pain) suggests that the underlying pathology is marked (Young, 1983; Khan et al., 1989).

The organs of the reproductive tract are considered immune privileged but do have a resident population of immune cells in the subepithelium of the epididymal duct and submucosa of the prostate acini (Foster, 2016). Infection with *B. melitensis* may stimulate an inflammatory response in these resident populations that leads to necrosis of the epididymal duct between 4 to 8-weeks post-infection. Spermatids contain unique genetic material which is recognized as “foreign” to immune cells; spermatids not contained within the lumen of the epididymal duct incite a strong inflammatory reaction (spermatic granuloma; Foster, 2016). Thus, inflammation of the reproductive tract associated with *B. melitensis* may not be correlated with current levels of colonization. Instead, it may indicate infection at an earlier time point. Intense inflammation in the epididymis can create an outflow obstruction, leading to spermiostasis and degeneration of the seminiferous tubules. Since the spermatic cycle in guinea pigs takes 2-weeks, infection with *B. melitensis* produced on-going spermiostasis in the guinea pig resulting in a paucity of mature spermatids and decreased fertility (Cleland, 1951). This suggests that fertility of infected men may still be negatively impacted in the absence of active infection.

Humans, mice, and guinea pigs have similar accessory sex glands (prostate, seminal vesicle) that contribute components of the seminal fluid to nourish the spermatozoa (Hargaden and Singer, 2012; Knoblaugh et al., 2018b). The prostate is a potential reservoir of *Brucella* spp., and infection of this organ in man can lead to abscesses and urinary tract infections (Boyd, 1938; Young, 1983). While a reservoir function cannot be appreciated due to the lack of detectable colonization at 8-weeks post-inoculation, previous infection of this organ in the guinea pig is reflected by an inflammatory response within the prostate which is similar to the lesions described in case reports of men with prostatitis (Savasci et al., 2014).

The stark contrast in protection afforded by the same vaccine in mice and guinea pigs emphasizes the necessity of evaluating candidates in more than one model. Several vaccine candidates have shown promising results in mice but have diminished efficacy when introduced into target species, like small ruminants (Carvalho et al., 2016). Guinea pigs are outbred animals and therefore may be more representative of vaccine efficacy than mouse models, which are often genetically homogeneous. An additional advantage of the guinea pig is the ability to evaluate both microbiological and histopathological results due to the larger size of the reproductive organs. Future experiments are required to determine optimal study end-points for vaccine efficacy in the guinea pig, but the results presented herein make a compelling case that guinea pigs are an appropriate animal model for evaluating the impact on the male reproductive tract.

## Conclusion

This study characterizes reproductive disease in two commonly available animal models. Understanding the pathogenesis of reproductive disease and evaluating potential vaccines for use in men requires an animal model that mimics the manifestation of human disease. This study demonstrates that infectious aerosols can generate reproductive disease in male guinea pigs and highlights the potential of intratracheal inoculation in guinea pigs to serve as a model for reproductive disease. Further studies are needed to evaluate vaccines in male animals and determine if the results presented herein are typical of vaccine efficacy in males.

## Data availability statement

The raw data supporting the conclusions of this article will be made available by the authors, without undue reservation.

## Ethics statement

The animal study was reviewed and approved by Institutional Animal Care and Use Committee Texas A&M University.

## Author contributions

MH, JE, and AA-G: conceptualized the project and performed data analysis. MH and LS: performed the experiments. MH: wrote the manuscript. All authors participated in manuscript review and approved the manuscript.

## Funding

Student stipend support was provided by the National Institutes of Health Institutional Training Grant T32 OD 11057 (MH).

## Conflict of interest

The authors declare that the research was conducted in the absence of any commercial or financial relationships that could be construed as a potential conflict of interest.

## Publisher’s note

All claims expressed in this article are solely those of the authors and do not necessarily represent those of their affiliated organizations, or those of the publisher, the editors and the reviewers. Any product that may be evaluated in this article, or claim that may be made by its manufacturer, is not guaranteed or endorsed by the publisher.
