# Supplementary material for: Intratracheal inoculation results in Brucella-associated reproductive disease in male mouse and guinea pig models of infection
Source: Front Microbiol. 2022 Oct 19;13:1029199. doi: 10.3389/fmicb.2022.1029199 (PMC9626965; doi:10.3389/fmicb.2022.1029199)
Supplement: Supplementary file 1 [file Table_1.DOCX]

| **Epididymis** | **Description** | **Score** |
| --- | --- | --- |
| Edema | High protein fluid in the interstitium | 0- absent |
|  |  | 1- present |
| Epididymal duct hyperplasia | Increased mitoses, piling of cells of the epididymal duct epithelium | 0- absent |
|  |  | 1- present |
| Inflammation of epididymis | Accumulation of histiocytic and neutrophilic inflammatory cells in the interstitium of the epididymal duct | 0- absent |
|  |  | 1- focal |
|  |  | 2- multifocal |
|  |  | 3- multifocal to coalescing |
|  |  | 4- diffuse |
| Necrosis of epididymal ducts | Loss of epididymal duct architecture and replacement by karyorrhectic debris surrounded by intense inflammation | 0- absent |
|  |  | 1- present |
| Spermatids in lumen of epididymal duct |  | 1- absent |
|  |  | 0- present |
| **Testicle** |  |  |
| Edema | High protein fluid in between seminiferous tubules | 0-absent |
|  |  | 1-present |
| Inflammation of testicle | Perivascular accumulation of lymphocytes and plasma cells | 0- absent |
|  |  | 1- absent |
| Spermatogenic arrest | Disordered progression of spermatogenesis | 0- absent |
|  |  | 1- present |
| Testicular degeneration | Multinucleated spermatids, decreased average size of testicle mass | 0- absent |
|  |  | 1- present |
| **Prostate** |  |  |
| Edema | High protein fluid in the interstitium | 0-absent |
|  |  | 1-present |
| Inflammation of prostate gland | Accumulation of histiocytic and neutrophilic inflammatory cells in the interstitium of the prostate gland | 0- absent |
|  |  | 1- focal |
|  |  | 2- multifocal |
|  |  | 3- multifocal to coalescing |
|  |  | 4- diffuse |
| Necrosis of prostate gland | Loss of prostate gland architecture and replacement by karyorrhectic debris surrounded by intense inflammation | 0- absent |
|  |  | 1- present |
